# Determinants of delayed or incomplete diphtheria-tetanus-pertussis vaccination in parallel urban and rural birth cohorts of 30,956 infants in Tanzania

**DOI:** 10.1186/s12879-019-3828-3

**Published:** 2019-02-26

**Authors:** Pranay Nadella, Emily R. Smith, Alfa Muhihi, Ramadhani A. Noor, Honorati Masanja, Wafaie W. Fawzi, Christopher R. Sudfeld

**Affiliations:** 1 Harvard College, Cambridge, MA USA; 2 Departments of Global Health and Population, Boston, MA USA; 3 Departments of Nutrition, Boston, MA USA; 4 Departments of Epidemiology, Harvard T.H. Chan School of Public Health, 665 Huntington Avenue, Boston, MA 02115 USA; 5 Africa Academy for Public Health, Dar es Salaam, Tanzania; 6 Ifakara Health Institute, Dar es Salaam, Tanzania

**Keywords:** Immunization, Vaccination, Child health, Rural, Urban, Tanzania

## Abstract

**Background:**

Delayed vaccination increases the time infants are at risk for acquiring vaccine-preventable diseases. Factors associated with incomplete vaccination are relatively well characterized in resource-limited settings; however, few studies have assessed immunization timeliness.

**Methods:**

We conducted a prospective cohort study examining Diphtheria-Tetanus-Pertussis (DTP) vaccination timing among newborns enrolled in a Neonatal Vitamin A supplementation trial (NEOVITA) conducted in urban Dar es Salaam (*n* = 11,189) and rural Morogoro Region (*n* = 19,767), Tanzania. We used log-binomial models to assess the relationship of demographic, socioeconomic, healthcare access, and birth characteristics with late or incomplete DTP1 and DTP3 immunization.

**Results:**

The proportion of infants with either delayed or incomplete vaccination was similar in Dar es Salaam (DTP1 11.5% and DTP3 16.0%) and Morogoro (DTP1 9.2% and DTP3 17.3%); however, the determinants of delayed or incomplete vaccination as well as their magnitude of association differed by setting. Both maternal and paternal education were more strongly associated with vaccination status in rural Morogoro region as compared to Dar es Salaam (*p*-values for heterogeneity < 0.05). Infants in Morogoro who had fathers and mothers with no education had 36% (95% CI: 22–52%) and 22% (95% CI: 10–34%) increased risk of delayed or incomplete DTP3 vaccination as compared to those with primary school education, respectively. In Dar es Salaam, mothers who attended their first antenatal care (ANC) visit in the 3rd trimester had 1.55 (95% CI: 1.36–1.78) times the risk of delayed or not received vaccination as compared to those with a 2nd trimester booking, while there was no relationship in Morogoro. In rural Morogoro, infants born at home had 17% (95% CI: 8–27%) increased risk for delayed or no receipt of DTP3 vaccination. In both settings, younger maternal age and poorer households were at increased risk for delayed or incomplete vaccination.

**Conclusion:**

We found some risk factors for delayed and incomplete vaccination were shared between urban and rural Tanzania; however, we found several context-specific risk factors as well as determinants that differed in their magnitude of risk between contexts. Immunization programs should be tailored to address context-specific barriers and enablers to improve timely and complete vaccination.

**Electronic supplementary material:**

The online version of this article (10.1186/s12879-019-3828-3) contains supplementary material, which is available to authorized users.

## Background

Routine childhood immunizations prevents an estimated 2 to 3 million deaths each year due to diphtheria, tetanus, pertussis, and measles [1]. Nevertheless, the maximal potential benefits of vaccines have not been realized in low- and middle-income countries (LMICs) due to incomplete coverage and late vaccination. The World Health Organization estimated that in 2015, over 19 million infants did not receive routine immunizations with the overwhelming majority coming from LMICs [1]. While suboptimal vaccine coverage in LMICs has been reasonably well documented, delayed vaccination has received significantly less attention. Delayed vaccination prolongs the at risk period and leaves infants vulnerable during early infancy, when the risk of severe morbidity or death is the greatest for most vaccine-preventable diseases [2].

Incomplete or delayed vaccination in LMICs may result from numerous factors including poor immunization supply chains, suboptimal access to health services, or social and cultural factors [3]. Several studies suggest that higher birth order, low maternal education, and low socioeconomic status are associated with poor adherence to vaccination schedules [3]. Studies in the Netherlands [4], Ghana [5] and Guinea-Bissau [6], have found that low birth weight was associated with delayed vaccination. In rural Ghana, greater distance to the health facility was also associated with delayed vaccination [5]. Nevertheless, no studies to the best of our knowledge have examined how the magnitude of the predictors of delayed vaccination vary by setting within the same country.

In this study, we analyzed parallel prospective cohorts of newborns in urban Dar es Salaam and rural Morogoro Region, Tanzania to assess potential differences in the contribution of social, economic, and biological factors to delayed or not received Diphtheria-Tetanus-Pertussis (DTP) vaccination. Through these comparisons, we intend to inform interventions to improve complete and timely vaccination in urban and rural immunization programs.

## Methods

### Study design and setting

This study utilized data from a randomized, double-blind controlled trial of Neonatal Vitamin A supplementation (NEOVITA). This trial enrolled 31,999 children in urban Dar es Salaam and rural Morogoro, Tanzania from August 2010 to March 2014 (ACTRN12610000636055). Briefly, newborns born at home or at a health facility were randomized to receive a mega-dose of vitamin A (50,000 IU) or placebo on the day of birth or within 3 days; trial recruitment and detailed data collection procedures have been presented elsewhere [7]. Newborns whose mothers were younger than 15 years old or older than 49 years were excluded from participation in the trial. In urban Dar es Salaam, 11,895 participants were enrolled at antenatal clinics and in labor wards of public health facilities in Kinondoni, Ilala, and Temeke districts. In Morogoro Region, 20,104 participants were recruited within a rural Health and Demographic Surveillance System (HDSS) that covers approximately 2400 km^2^ and allowed for enrollment of both health facility and home births in Kilombero, Ulanga and Kilosa districts.

### Enrollment and data collection

Newborns were eligible for randomization if they were able to feed orally, were born within the past three days, were not previously enrolled in other clinical trials, intended to stay in the study area for at least six months post-delivery, and the parents provided written informed consent to participate. At the baseline visit, trained study staff administered a baseline questionnaire to mothers in order to collect information on demographic, socioeconomic, and health factors. Trained fieldworkers visited the infants 1 and 3 days after enrollment, as well as 1, 3, 6, and 12 months after birth. During these visits, fieldworkers assessed the morbidity and mortality of the infants and ascertained information about the infants’ recent nutrition intake and any immunizations the infant received since the last visit [7, 8]. All newborns enrolled in the trial were given a child health card at the time of randomization. DTP vaccination status was determined by field workers who reviewed the child health card recorded dates of vaccination at each study visit (1 day, 3 days, and then 1, 3, 6, and 12 months). Child health cards were not available at less than 0.1% of all study visits and in these few cases the date of vaccination was reported by the mother or infant caregiver.

### Definition of delayed vaccination and predictors

In this study, we analyzed DTP1 and DTP3 vaccination timing. Tanzania Mainland recommended DTP1 vaccination at 4 weeks of age; however, during the trial in 2012, the pentavalent DTP + Haemophilus influenza type B (Hib) + Hepatitis B (HepB) vaccine was introduced and the recommended age at vaccination was increased to 6 weeks [9]. Tanzania recommended DTP3 at 14 weeks of age throughout the study.

There is no universal definition of delayed vaccination for DTP vaccine, although most, including the Pan American Health Organization, define late vaccination as one month or more after the recommended age of vaccination [10]. At the time of the study, LMICs were advised to set their recommended age at DTP1 vaccination at 6 weeks (4 weeks–2 months) [11, 12]. As a result, in the primary analysis we define delayed DTP1 vaccination as receipt > 90 days of age based on the upper recommended age for DTP1 (2 months or 60 days) plus 30 days late. In a sensitivity analysis, we define delayed vaccination as > 72 days based on the Tanzanian recommended age for the first pentavalent vaccine dose (6 weeks or 42 days) plus 30 days late. Due to the staggered roll-out of the pentavalent vaccine program during the study, we were not able to determine if infants were due for their first DTP vaccination at 4 weeks or 6 weeks and therefore we only present the conservative definition of 72 days late in the sensitivity analysis. Infants who were lost to follow up, died or were vaccinated before 15 days were excluded from the DTP1 analysis. The standard recommended age range for DTP3 vaccination in LMICs was 14 weeks [12 weeks–6 months]) [11, 12]. As a result, we defined delayed DTP3 vaccination as receipt > 210 days (7 months) of age in the main analysis. In a sensitivity analysis, we defined delayed DTP3 vaccination as > 128 days of age based on the Tanzanian recommended age of 14 weeks (98 days) plus 30 days late. Infants who were lost to follow up, died or were vaccinated before 60 days were excluded from the DTP3 analysis.

### Statistical analysis

Univariate and multivariate relative risks of delayed or incomplete DTP1 and DTP3 vaccination (> 90 days and > 210 days, respectively) were calculated using log-binomial models stratified by Dar es Salaam and Morogoro region [13]. We also present sensitivity analyses using delayed vaccination definitions of > 72 days and > 128 days for DTP1 and DTP3, respectively. Log-binomial models did not converge in a few instances and in these cases log-Poisson models, which provide consistent but not fully efficient estimates of the relative risk and its confidence intervals, were used [14]. Preterm birth was defined as delivery at < 37 weeks gestation as assessed by maternal report of last menstrual period. Small-for-gestational-age (SGA) was defined, with the use of Oken standards, as birth weight < 10th percentile for gestational age and sex [15]. A wealth index was generated based on household ownership of assets, and households were categorized into wealth quintiles stratified by Dar es Salaam and Morogoro residence [16]. Due to collinearity, low birth weight was modeled separately from preterm birth and small-for-gestational age. *P*-values for trend in categorical analyses were calculated by treating the median value of each category as a continuous variable. The log-rank test was used to assess the statistical significance of potential effect modification of predictors of interest by study site (Dar es Salaam versus Morogoro). Missing data were retained with use of the missing indicator method. All *P* values were 2-sided with a *P* <  0.05 considered statistically significant. All of the analyses for this study were conducted using R version 3.3.1.

## Results

In the parent NEOVITA trial, 32,483 (96.3%) newborns were screened for study eligibility and 31,999 (97.43%) were enrolled. The analysis of DTP1 and DTP3 included the 30,956 infants and 30,503 infants, respectively, who did not die and were not lost to follow-up before the start of the vaccination window. A participant flowchart is included in Additional file 1: Figure S1. Table 1 presents baseline characteristics of the urban Dar es Salaam and rural Morogoro study cohorts. The Dar es Salaam cohort had a lower proportion of low birth weight infants (8.7%) as compared to rural Morogoro (13.9%). In Dar es Salam 9.3% of infants were born SGA, while 13.6% were SGA in Morogoro. Infants from Morogoro were also of higher parity, with 17.7% being the 5th or higher child, as compared to 8.1% in Dar es Salaam. Mothers in Dar es Salaam were slightly more likely to attend their first antenatal care visit in the first trimester (13.3%) than mothers in Morogoro (9.6%). In addition, 13.4% of births occurred in the home in rural Morogoro, while there was a negligible amount (< 1%) of home births in the Dar es Salaam cohort. Mothers and fathers in Dar es Salaam were more likely to have completed school beyond the primary level and less likely to have had no formal schooling than mothers and fathers in Morogoro.

**Table 1:** Characteristics of participants of vaccination study cohort stratified by urban Dar es Salaam (*n* = 11,189) and rural Morogoro (*n* = 19,767) site

Characteristic	Urban Dar es Salaam N = 11,189 n (%)	Rural Morogoro Region N = 19,767 n (%)
Infant sex		
Male	5712 (51.1%)	10,517 (53.2%)
Female	5473 (48.9%)	9250 (46.8%)
Parity		
Firstborn	2577 (30.3%)	4865 (28.7%)
2nd-4th child	5239 (61.6%)	9083 (53.6%)
5th or greater child	686 (8.1%)	3006 (17.7%)
Birthweight		
Low birthweight (< 2500 g)	967 (8.7%)	2741 (13.9%)
Normal birthweight (≥2500 g)	10,208 (91.3%)	17,026 (86.1%)
Gestational Age		
Preterm (< 37 weeks)	1511 (19.0%)	1887 (15.9%)
Term (≥37 weeks)	6430 (81.0%)	9978 (84.1%)
Size for gestational Age		
SGA (<10th percentile)	729 (9.3%)	1572 (13.6%)
No SGA (≥10th percentile)	7084 (90.7%)	9987 (86.4%)
Maternal age		
< 20 years	1835 (18.5%)	4517 (23.6%)
20–25 years	3269 (33.0%)	5266 (27.5%)
25–30 years	2825 (28.5%)	5256 (27.4%)
30–35 years	1396 (14.1%)	2744 (14.3%)
≥ 35 years	589 (5.9%)	1397 (7.3%)
Maternal education		
No formal schooling	466 (4.8%)	2098 (10.9%)
Some primary	347 (3.6%)	1872 (9.8%)
Completed primary	7291 (74.7%)	13,974 (72.9%)
Secondary plus	1656 (17.0%)	1225 (6.4%)
Paternal education		
No formal schooling	189 (1.9%)	1218 (6.4%)
Some primary	221 (2.2%)	1481 (7.7%)
Completed primary	6631 (67.8%)	14,588 (76.1%)
Secondary plus	2742 (28.0%)	1889 (9.9%)
Place of birth		
Home	60 (0.6%)	2646 (13.4%)
Facility	10,819 (99.4%)	17,100 (86.6%)
Trimester of first ANC visit		
1st Trimester	1133 (13.3%)	1536 (9.6%)
2nd Trimester	6604 (77.3%)	12,486 (77.8%)
3rd Trimester	802 (9.4%)	2031 (12.7%)

**Fig. 1:**
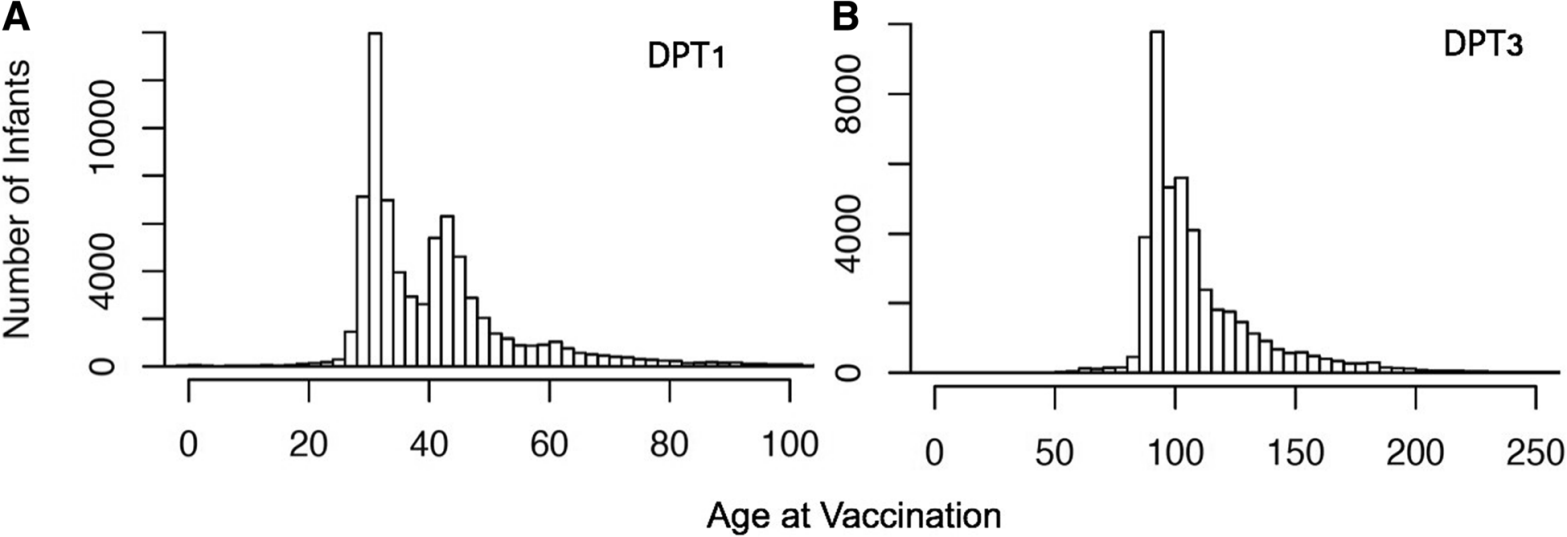
Histogram of age at DTP1 (Panel **a**) and DTP3 (Panel **b**) vaccination for infants in Morogoro and Dar es Salaam regions

In Dar es Salaam, of 11,189 infants eligible for DTP1 analysis, 9906 (88.5%) were vaccinated on time, 336 (3.0%) were vaccinated late (> 90 days), and 947 (8.5%) were not observed to be vaccinated during follow-up to 1 year of age. Of 10,932 infants eligible for DTP3 analysis in Dar, 9181 (84.0%) were vaccinated on time, 200 (1.8%) were vaccinated late (> 7 months), and 1551 (14.2%) were not observed to be vaccinated to 1 year of age. In Morogoro Region, of 19,767 infants eligible for DTP1 analysis, 17,959 (90.9%) were vaccinated on time, 370 (1.9%) were vaccinated late, and 1439 (7.3%) were never vaccinated. Of 19,571 infants eligible for DTP3 analysis in Morogoro Region, 16,190 (82.7%) were vaccinated on time, 516 (2.6%) were vaccinated late, and 2865 (14.6%) were never vaccinated. The distribution of infant age (days) at vaccination with DTP1 and DTP3 by site are presented in Fig. 1. The bimodal shape of the DTP1 vaccination age in Fig. 1a is due to an increase in the Tanzanian recommended age at the first DTP dose from 4 weeks to 6 weeks with the introduction of the pentavalent vaccine. The shape of the DTP3 age histogram is monomodal since the recommended age at DTP3 vaccination was 14 weeks both before and after introduction of the pentavalent vaccine.

Risk factors associated with delayed or incomplete DTP1 and DTP3 vaccination are presented in Tables 2 and 3. We determined that there was no difference in risk by infant sex of delayed or no receipt of DTP1 or DTP3 vaccination in both urban Dar es Salaam and rural Morogoro cohorts. The association between low birth weight and delayed or incomplete DTP1 vaccination varied by study cohort (*p*-value for interaction 0.009). In Morogoro region, low birthweight infants had 31% (95% CI: 20–40%) and 29% (95% CI: 21–36%) reduced risk of delayed or not received DTP1 and DTP3 vaccination, respectively. There was no significant association of low birth weight with DTP vaccination in Dar es Salaam. There was also no relationship between prematurity or small-for-gestational-age births and vaccination timing in either setting.

**Table 2:** Predictors of late (> 3 months of age) or no receipt of DTP1 vaccination among urban Dar es Salaam (n = 11,189) and rural Morogoro (n = 19,767) infants

Characteristic	Urban Dar es Salaam				Rural Morogoro Region				p-value for interaction by site
	Total N	Late or no receipt of DTP1 n (%)	Multivariate Relative Risk (95% CI)	p value	Total N	Late or no receipt of DTP1 n (%)	Multivariate Relative Risk (95% CI)	*p* value	
Total cohort	11,189	1283 (11.47%)	–	–	19,767	1808 (9.15%)	–	–	
Infant sex									
Male	5712	669 (11.71%)	1.04 (0.94–1.16)	0.40	10,517	983 (9.35%)	1.05 (0.96–1.15)	0.26	0.93
Female	5473	613 (11.20%)	Reference		9250	826 (8.93%)	Reference		
Parity									
Firstborn	2577	332 (12.88%)	Reference		4865	514 (10.57%)	Reference		0.17
2nd-4th child	5239	566 (10.80%)	0.95 (0.82–1.10)	0.48	9083	808 (8.90%)	0.83 (0.73–0.94)	0.003	
5th or greater child	686	80 (11.66%)	1.05 (0.81–1.35)	0.73	3006	330 (10.98%)	1.05 (0.89–1.25)	0.53	
Birthweight									
Low birthweight (< 2500 g)	967	113 (11.69%)	0.95 (0.79–1.14)	0.56	2741	184 (6.71%)	0.69 (0.60–0.80)	< 0.001	0.009
Normal birthweight (≥2500 g)	10,208	1167 (11.43%)	Reference		17,026	1625 (9.54%)	Reference		
Gestational Age									
Preterm (< 37 weeks)	1511	172 (11.38%)	1.00 (0.85–1.17)	0.97	1887	189 (10.02%)	0.99 (0.85–1.16)	0.91	0.60
Term (≥37 weeks)	6430	674 (10.48%)	Reference		9978	898 (9.00%)	Reference		
Size for gestational Age									
SGA (<10th percentile)	729	77 (10.56%)	0.88 (0.71–1.11)	0.28	1572	134 (8.52%)	0.93 (0.78–1.11)	0.40	0.76
No SGA (≥10th percentile)	7084	755 (10.66%)	Reference		9987	918 (9.19%)	Reference		
Maternal age									
< 20 years	1835	247 (13.46%)	1.48 (1.23–1.78)	< 0.001*	4517	446 (9.87%)	1.00 (0.86–1.15)	0.003*	0.23
20–25 years	3269	340 (10.40%)	1.18 (1.01–1.39)		5266	513 (9.74%)	1.08 (0.96–1.22)		
25–30 years	2825	247 (8.74%)	Reference		5256	478 (9.09%)	Reference		
30–35 years	1396	121 (8.67%)	0.96 (0.78–1.18)		2744	208 (7.58%)	0.82 (0.70–0.96)		
≥ 35 years	589	54 (9.17%)	0.98 (0.73–1.31)		1397	107 (7.66%)	0.77 (0.62–0.94)		
Maternal education									
No formal schooling	466	58 (12.45%)	1.16 (0.90–1.50)	0.05*	2098	264 (12.58%)	1.13 (0.98–1.30)	0.17*	0.29
Some primary	347	43 (12.39%)	1.09 (0.82–1.45)		1872	203 (10.84%)	1.10 (0.95–1.28)		
Completed primary	7291	746 (10.23%)	Reference		13,974	1169 (8.37%)	Reference		
Secondary plus	1656	144 (8.70%)	0.89 (0.74–1.06)		1225	107 (8.73%)	1.11 (0.91–1.35)		
Paternal education									
No formal schooling	189	20 (10.58%)	0.88 (0.58–1.35)	0.14*	1218	197 (16.17%)	1.59 (1.36–1.87)	< 0.001*	0.04
Some primary	221	38 (17.19%)	1.57 (1.16–2.11)		1481	177 (11.95%)	1.32 (1.13–1.53)		
Completed primary	6631	686 (10.35%)	Reference		14,588	1228 (8.42%)	Reference		
Secondary plus	2742	243 (8.86%)	0.93 (0.80–1.08)		1889	139 (7.36%)	0.93 (0.78–1.11)		
Wealth quintile									
Q1 (Poorest)	1577	208 (13.12%)	1.43 (1.13–1.80)	0.01*	3988	433 (10.86%)	1.32 (1.13–1.54)	< 0.001*	0.02
Q2	2325	232 (9.98%)	1.12 (0.89–1.41)		4350	488 (11.22%)	1.35 (1.16–1.57)		
Q3	1712	183 (10.69%)	1.24 (0.98–1.57)		3247	240 (7.39%)	1.04 (0.88–1.23)		
Q4	2988	283 (9.47%)	1.15 (0.92–1.42)		3810	318 (8.35%)	1.13 (0.97–1.32)		
Q5 (Richest)	1282	102 (7.96%)	Reference		3828	276 (7.21%)	Reference		
Place of birth^a^									
Home	–	–	–	–	2646	310 (11.72%)	1.20 (1.06–1.35)	0.004	
Facility	–	–	–		17,100	1492 (8.73%)	Reference		
Trimester of first ANC visit									
1st Trimester	1133	112 (9.89%)	0.86 (0.71–1.04)	< 0.001*	1536	144 (9.38%)	0.90 (0.76–1.06)	0.63*	0.001
2nd Trimester	6604	737 (11.16%)	Reference		12,486	1259 (10.08%)	Reference		
3rd Trimester	802	139 (17.33%)	1.55 (1.32–1.83)		2031	196 (9.65%)	0.95 (0.83–1.10)		

*p value for trend

^a^Participants in Dar es Salaam primarily enrolled from facilities

**Table 3:** Predictors of late (> 7 months of age) or no receipt of DTP3 vaccination among urban Dar es Salaam (*n* = 10,932) and rural Morogoro (*n* = 19,571) infants

Characteristic	Urban Dar es Salaam				Rural Morogoro Region				p-value for interaction by site
	Total N	Late or no receipt of DTP3 n (%)	Multivariate Relative Risk (95% CI)	p value	Total N	Late or no receipt of DTP3 n (%)	Multivariate Relative Risk (95% CI)	p value	
Total cohort	10,932	1751 (16.01%)	–	–	19,571	3381 (17.28%)	–	–	
Infant sex									
Male	5578	919 (16.48%)	1.06 (0.97–1.15)	0.20	10,406	1801 (17.31%)	1.01 (0.95–1.07)	0.77	0.29
Female	5350	831 (15.53%)	Reference		9165	1580 (17.24%)	Reference		
Parity									
Firstborn	2509	423 (16.86%)	Reference		4807	886 (18.43%)	Reference		0.02
2nd-4th child	5147	807 (15.68%)	1.10 (0.98–1.25)	0.11	8989	1519 (16.90%)	0.91 (0.84–1.00)	0.04	
5th or greater child	668	103 (15.42%)	1.19 (0.95–1.48)	0.13	2978	638 (21.42%)	1.18 (1.05–1.32)	0.006	
Birthweight									
Low birthweight (< 2500 g)	942	139 (14.76%)	0.85 (0.73–1.00)	0.050	2713	342 (12.61%)	0.71 (0.64–0.79)	< 0.001	0.06
Normal birthweight (≥2500 g)	9990	1612 (16.14%)	Reference		16,858	3039 (18.03%)	Reference		
Gestational Age									
Preterm (< 37 weeks)	1478	244 (16.51%)	1.01 (0.89–1.16)	0.84	1861	340 (18.27%)	0.91 (0.82–1.01)	0.09	0.72
Term (≥37 weeks)	6280	928 (14.78%)	Reference		9887	1772 (17.92%)	Reference		
Size for gestational Age									
SGA (<10th percentile)	710	106 (14.93%)	0.90 (0.75–1.08)	0.26	1559	256 (16.42%)	0.92 (0.82–1.04)	0.18	0.55
No SGA (≥10th percentile)	6922	1041 (15.04%)	Reference		9885	1789 (18.10%)	Reference		
Maternal age									
< 20 years	1785	347 (19.44%)	1.58 (1.36–1.84)	< 0.001*	4472	836 (18.69%)	1.10 (1.00–1.21)	< 0.001*	0.02
20–25 years	3204	481 (15.01%)	1.20 (1.05–1.36)		5219	942 (18.05%)	1.11 (1.02–1.21)		
25–30 years	2783	356 (12.79%)	Reference		5196	865 (16.65%)	Reference		
30–35 years	1378	165 (11.97%)	0.90 (0.76–1.07)		2725	429 (15.74%)	0.92 (0.82–1.02)		
≥ 35 years	579	64 (11.05%)	0.81 (0.62–1.04)		1384	220 (15.90%)	0.85 (0.74–0.98)		
Maternal education									
No formal schooling	655	105 (16.03%)	1.00 (0.83–1.21)	0.21*	2175	520 (23.91%)	1.22 (1.10–1.34)	< 0.001*	0.049
Some primary	339	60 (17.70%)	1.05 (0.83–1.33)		1858	416 (22.39%)	1.25 (1.13–1.38)		
Completed primary	7163	1062 (14.83%)	Reference		13,847	2185 (15.78%)	Reference		
Secondary plus	1625	195 (12.00%)	0.86 (0.74–1.00)		1213	177 (14.59%)	0.99 (0.85–1.15)		
Paternal education									
No formal schooling	187	26 (13.90%)	0.84 (0.58–1.22)	0.05*	1197	339 (28.32%)	1.36 (1.22–1.52)	< 0.001*	0.004
Some primary	217	56 (25.81%)	1.66 (1.31–2.10)		1461	316 (21.63%)	1.18 (1.06–1.31)		
Completed primary	6495	965 (14.86%)	Reference		14,459	2360 (16.32%)	Reference		
Secondary plus	2702	336 (12.44%)	0.92 (0.81–1.04)		1887	261 (13.91%)	0.96 (0.85–1.09)		
Wealth quintile									
Q1 (Poorest)	1544	300 (19.43%)	1.25 (1.04–1.49)	0.006*	3938	807 (20.49%)	1.32 (1.18–1.47)	< 0.001*	< 0.001
Q2	2276	318 (13.97%)	0.93 (0.78–1.11)		4311	926 (21.48%)	1.37 (1.23–1.52)		
Q3	1672	250 (14.95%)	1.03 (0.86–1.23)		3222	447 (13.87%)	1.01 (0.89–1.13)		
Q4	2947	374 (12.69%)	0.91 (0.77–1.08)		3769	584 (15.49%)	1.10 (0.98–1.23)		
Q5 (Richest)	1258	169 (13.43%)	Reference		3793	514 (13.55%)	Reference		
Place of birth									
Home^a^	–	–	–	–	2614	584 (22.34%)	1.17 (1.08–1.27)	< 0.001	
Facility	–	–	–		16,938	2789 (16.47%)	Reference		
Trimester of first ANC visit									
1st Trimester	1108	158 (14.26%)	0.92 (0.79–1.07)	< 0.001*	1517	261 (17.21%)	0.89 (0.79–0.99)	0.19*	< 0.001
2nd Trimester	6469	996 (15.40%)	Reference		12,350	2288 (18.53%)	Reference		
3rd Trimester	781	187 (23.94%)	1.55 (1.36–1.78)		2014	370 (18.37%)	0.98 (0.89–1.09)		

*p value for trend

^a^Participants in Dar es Salaam primarily enrolled from facilities

Infants in the highest wealth quintile had a reduced risk of delayed or incomplete vaccination with DTP1 and DTP3 in both Dar es Salaam and Morogoro. This association between wealth quintile and delayed or incomplete vaccination was stronger in Morogoro than in Dar es Salaam for both DTP1 and DTP3 (*p*-values for interaction 0.02, < 0.001). The magnitude of the association between paternal education and delayed or incomplete vaccination was stronger in Morogoro for both DTP1 and DTP3 (p-value for interaction 0.04, 0.004). In rural Morogoro, infants of fathers with no formal education had a 59% (95% CI: 36, 87%) and 36% (95% CI: 22, 52%) increased risk of delayed or not received DTP1 and DTP3 vaccination, respectively as compared to those whose fathers completed primary school. In contrast, there was no indication that infants of non-educated fathers had an increased risk of delayed or not received vaccination in Dar es Salaam. Greater maternal education was significantly associated with reduced risk of delayed or incomplete vaccination with DTP3 in Morogoro (*p*-value < 0.001), but there was no significant association of maternal education with DTP3 vaccination in Dar es Salaam.

In terms of indicators of access to care, mothers in Dar es Salaam who attended their first ANC visit later in pregnancy had an increased risk of delayed or no receipt of both DTP1 (p-value < 0.001) and DTP3 (p-value < 0.001) vaccination. There was no relationship of ANC timing and vaccination status in rural Morogoro. Nevertheless, home births in the Morogoro Region had 1.20 (95% CI: 1.06, 1.35) and 1.17 (95% CI: 1.08, 1.27) times the risk of delayed or incomplete DTP1 and DTP3 vaccination as compared to facility births, respectively.

As expected, the percentage of infants with delayed vaccination increased when the earlier cut-offs of > 72 days for DTP1 and > 128 days for DTP3 were used in sensitivity analyses (Additional file 1: Tables S1 and S2). For DTP1, 15.3 and 11.8% of infants had delayed or incomplete vaccination in Dar es Salaam and Morogoro, respectively. For DTP3 vaccination, these percentages were 31.5 and 33.6%, respectively. In terms of risk factors, we found similar effect sizes in the sensitivity analysis (Additional file 1: Tables S1 and S2) and that the magnitude of the associations for wealth, paternal education, low birthweight, and ANC timing differed between Dar es Salaam and Morogoro Region.

## Discussion

In this prospective birth cohort study of 30,956 infants from urban Dar es Salaam and rural Morogoro Region, Tanzania, we determined that the proportion of infants with incomplete or late vaccination was similar between the contexts at about 10% for DTP1 and 15% for DTP3. We found some shared determinants of delayed or incomplete vaccination between settings; however, several factors differed in magnitude between urban and rural settings. In urban Dar es Salaam, infants born to young mothers, infants from households in the lowest wealth quintile, and mothers whose first ANC visit was in the third trimester were more likely to be vaccinated late or never. In rural Morogoro region, infants born normal birth weight, at home, to uneducated fathers, to poorer households, and to young mothers had an elevated risk of delayed or incomplete DTP vaccination.

Infants born to wealthier households were less likely to experience delayed or incomplete vaccination in both urban and rural settings in our study. The association between greater household wealth and reduced risk of delayed or incomplete vaccination has been previously noted [3]. Access to greater financial resources may address the costs associated with vaccination, including transport to the healthcare facility and parents foregoing daily wage to accompany their child to health clinics. Furthermore, individuals in the lowest wealth quintiles are more likely to live further from healthcare facilities [17], which is a commonly reported driver of poor vaccination uptake [18]. We did not have access to data on the distance to the nearest health center in our study. Financial and non-financial incentives to improve vaccination coverage have shown mixed effectiveness through extensive trials in LMICs [19, 20]. As a result, further implementation studies and trials are needed to determine effective interventions to overcome health care access barriers to vaccination among urban and rural poor families.

Younger maternal age was associated with delayed or incomplete vaccination with DTP3 in both Dar es Salaam and Morogoro region. However, lower maternal education was associated with an increased risk of delayed or incomplete vaccination with DTP3 in rural Morogoro, but there was no evidence of an association in Dar es Salaam. Strong relationships between maternal education, maternal age, and vaccination status have been observed in both high-income and LMIC settings, including Tanzania [3, 21]. We also found that lower paternal education, independent of maternal education, was associated with an increased risk of delayed or no receipt of DTP vaccination in both Dar es Salaam and Morogoro; however, the magnitude of the association was significantly stronger in rural Morogoro. Research has shown that lack of support by men can inhibit access of women to family planning and healthcare, particularly when men hold greater economic power within the households [22, 23]. Our data suggest that paternal sensitization and involvement interventions may be effective in improving vaccine uptake in rural Tanzania and similar settings, but these interventions may not as effective in improving vaccination timeliness in urban centers.

Low birthweight was associated with reduced risk of delayed or not received DTP1 and DTP3 vaccination in rural Morogoro Region, while there was no relationship in urban Dar es Salaam. This result is the opposite of findings from both high-income countries, like the Netherlands [4], and low-income countries, like Ghana [5], Guinea-Bissau [6], and Kenya [24], where low birthweight was associated with increased risk of delayed or incomplete vaccination. Preterm birth has also been shown to be associated with delayed vaccination in high-income settings [18]. In some LMIC settings, caregivers may be reluctant to bring ill or undernourished children to a clinic for vaccination due to social stigma and providers may delay vaccination of ill children [18]. Nevertheless, in rural Tanzania we found low birth weight infants were significantly less likely to have delayed or incomplete vaccinations. While some LMICs recommend waiting until infants have reached desired weights before administering certain vaccines [24], Tanzania’s Expanded Program for Immunization (EPI) does not do so [9]. Targeting low birth weight or preterm infants with additional counselling on vaccination does not appear to be a promising intervention to improve vaccination status in the study context.

Home birth was strongly associated with increased risk of delayed or no receipt of DTP vaccination in Morogoro region while third-trimester antenatal care visit was associated with an increased risk in Dar es Salaam. Distance to a healthcare facility is known to be a primary barrier to antenatal care, facility births, and timely childhood vaccination [18, 25, 26]. Furthermore, decreased health seeking behavior caused by negative perceptions of formal healthcare has also been shown to contribute to home births, poor antenatal care use, and delayed vaccination [26–28]. However, there has not been widespread documentation of negative perceptions of routine child health services and vaccination in Tanzania. A qualitative study in rural Morogoro region has documented strong positive perception and high value of vaccination in the community [29]. Accordingly, interventions that target homebirths and improve healthcare system access in rural Tanzania and counselling interventions for women who book ANC late may significantly improve vaccination rates in urban Tanzania.

This study has several limitations. First, while the rural Morogoro region sample was a population-representative sample of all facility and home births within a demographic surveillance system, the Dar es Salaam site only recruited newborns born in a facility which may somewhat limit its representativeness. Nevertheless, the 2016 Tanzanian Demographic and Health Survey (DHS) found that 94% of women in Dar es Salaam delivered at a health facility and as a result the relatively small number of home births in this context would likely have minimal impact on our risk estimates [30]. The parent trial excluded infants in the first 3 days of life who were not able to feed orally. As a result, our study is not able to examine vaccination status among these high-risk infants; however, only 38 newborns were excluded for this reason in the parent trial and therefore would have minimal to no effect on our risk factor estimates. In addition, we did not collect information on the distance between home and the nearest health facility, which is known to influence the risk of delayed vaccination, particularly in rural areas [31]. Furthermore, in this study, fieldworkers conducted home visits primarily to assess infant vital status which may have had some impact on care-seeking behaviors; however, fieldworkers were not trained to counsel on vaccination timeliness and therefore the effect on delayed and no receipt of DTP vaccination is likely minimal. Nevertheless, there are several major strengths to this study. It is the largest birth cohort study of vaccination timing containing both an urban and rural sample. Furthermore, our prospective cohort design allowed us to accurately ascertain vaccination status and timing among infants who died during follow-up. Coverage and risk estimates from cross-sectional vaccination surveys, like the DHS, can be prone to survival bias since vaccination status is difficult to obtain post mortem as the vaccination card may be destroyed, mothers may travel after a child dies, or fieldworkers may be reluctant to ask for vaccination cards [32]. In addition, we prospectively followed infants from birth, which unlike cross-sectional studies, provides complete and accurate data on vaccination timing and birth risk factors.

## Conclusions

We found that both urban and rural infants were at risk for not being vaccinated on time. While some of the risk factors for delayed or incomplete vaccination appear to be  important in both urban and rural settings, others varied significantly by context. Our data strongly suggests that national immunization programs should tailor interventions to the local context. In both urban Dar es Salaam and rural Morogoro Region, interventions targeting poor households, young mothers, and mothers with low education may decrease delayed or incomplete vaccination. However, in Morogoro Region, additional interventions focused on home births and fathers with low education appear to be particularly promising to improve timely vaccination. Future randomized trials and rigorous implementation evaluations should determine the effectiveness of intervention packages and strategies to address barriers to timely immunization in diverse urban and rural African settings.

## Additional file

Additional file 1: **Figure S1.** and **Tables S1.** and **S2.** Participant flow chart and sensitivity analyses. (DOCX 97 kb)

## Abbreviations

ANC: Antenatal Care
DTP: Diphtheria-Tetanus-Pertussis
HepB: Hepatitis B
Hib: *Haemophilus influenzae* type B
LBW: Low birthweight
LMIC: Low- and middle-income country
NEOVITA: Neonatal Vitamin A Supplementation trial
RR: Relative risk
SGA: Small-for-gestational-age
