# Supplementary material for: Determinants of delayed or incomplete diphtheria-tetanus-pertussis vaccination in parallel urban and rural birth cohorts of 30,956 infants in Tanzania
Source: BMC Infect Dis. 2019 Feb 26;19:188. doi: 10.1186/s12879-019-3828-3 (PMC6390320; doi:10.1186/s12879-019-3828-3)
Supplement: Supplementary file 1 — Figure S1. and Tables S1. and S2. Participant flow chart and sensitivity analyses. (DOCX 97 kb) [file 12879_2019_3828_MOESM1_ESM.docx]

Additional file 1: Figure S1. Identification, screening and enrollment in the NEOVITA trial in Tanzania as well as inclusion and definitions for the DTP1 and DTP3 analyses.

Table S1. Sensitivity Analysis for DTP1 - Predictors of late (> 72 days of age) or no receipt of DTP1 vaccination among urban Dar es Salaam (n=11,189) and rural Morogoro (n=19,767) infants.

|  | Urban Dar es Salaam | | | | Rural Morogoro Region | | | | p-value for interaction by site |
| --- | --- | --- | --- | --- | --- | --- | --- | --- | --- |
| Characteristic | Total N | Late or no receipt of DTP1  n (%) | Multivariate Relative Risk (95% CI) | p value | Total N | Late or no receipt of DTP1  n (%) | Multivariate Relative Risk (95% CI) | p value |  |
| Total cohort | 11189 | 1709 (15.27%) | - | - | 19767 | 2328 (11.78%) | - | - |  |
|  |  |  |  |  |  |  |  |  |  |
| Infant sex |  |  |  |  |  |  |  |  |  |
| Male | 5712 | 892 (15.62%) | 1.05 (0.96 – 1.14) | 0.32 | 10517 | 1256 (11.94%) | 1.04 (0.96 – 1.12) | 0.34 | 0.72 |
| Female | 5473 | 815 (14.89%) | Reference |  | 9250 | 1072 (11.59%) | Reference |  |  |
| Parity |  |  |  |  |  |  |  |  |  |
| Firstborn | 2577 | 434 (16.84%) | Reference |  | 4865 | 636 (13.07%) | Reference |  | 0.32 |
| 2^nd^-4^th^ child | 5239 | 773 (14.75%) | 0.98 (0.87 – 1.11) | 0.77 | 9083 | 1023 (11.26%) | 0.83 (0.75 – 0.93) | < 0.001 |  |
| 5^th^ or greater child | 686 | 115 (16.76%) | 1.11 (0.90 – 1.38) | 0.34 | 3006 | 446 (14.84%) | 1.07 (0.93 – 1.24) | 0.35 |  |
| Birthweight |  |  |  |  |  |  |  |  |  |
| Low birthweight (<2500g) | 967 | 153 (15.82%) | 0.98 (0.84 – 1.13) | 0.75 | 2741 | 253 (9.23%) | 0.75 (0.66 – 0.86) | < 0.001 | 0.01 |
| Normal birthweight (≥2500g) | 10208 | 1553 (15.21%) | Reference |  | 17026 | 2075 (12.19%) | Reference |  |  |
| Gestational Age |  |  |  |  |  |  |  |  |  |
| Preterm (<37 weeks) | 1511 | 227 (15.02%) | 1.01 (0.88 – 1.16) | 0.90 | 1887 | 260 (13.78%) | 1.04 (0.91 – 1.18) | 0.56 | 0.17 |
| Term (≥37 weeks) | 6430 | 896 (13.93%) | Reference |  | 9978 | 1190 (11.93%) | Reference |  |  |
| Size for gestational Age |  |  |  |  |  |  |  |  |  |
| SGA (<10th percentile) | 729 | 113 (15.50%) | 1.01 (0.84 – 1.21) | 0.91 | 1572 | 193 (12.28/%) | 1.03 (0.89 – 1.19) | 0.40 | 0.58 |
| No SGA (≥10th percentile) | 7084 | 990 (13.98%) | Reference |  | 9987 | 1211 (12.13%) | Reference |  |  |
| Maternal age |  |  |  |  |  |  |  |  |  |
| <20 years | 1835 | 329 (17.93%) | 1.44 (1.23 – 1.69) | < 0.001* | 4517 | 568 (12.57%) | 1.03 (0.91 – 1.17) | 0.003* | 0.31 |
| 20-25 years | 3269 | 465 (14.22%) | 1.18 (1.04 – 1.35) |  | 5266 | 638 (12.12%) | 1.09 (0.98 – 1.21) |  |  |
| 25-30 years | 2825 | 341 (12.07%) | Reference |  | 5256 | 592 (11.26%) | Reference |  |  |
| 30-35 years | 1396 | 174 (12.46%) | 1.00 (0.84 – 1.18) |  | 2744 | 293 (10.68%) | 0.92 (0.81 – 1.06) |  |  |
| ≥ 35 years | 589 | 82 (13.92%) | 1.06 (0.84 – 1.35) |  | 1397 | 161 (11.52%) | 0.91 (0.77 – 1.08) |  |  |
| Maternal education |  |  |  |  |  |  |  |  |  |
| No formal schooling | 466 | 78 (16.74%) | 1.11 (0.90 – 1.38) | 0.06* | 2098 | 352 (16.78%) | 1.19 (1.05 – 1.34) | 0.004* | 0.37 |
| Some primary | 347 | 59 (17.00%) | 1.09 (0.86 – 1.38) |  | 1872 | 275 (14.69%) | 1.17 (1.03 – 1.33) |  |  |
| Completed primary | 7291 | 1033 (14.17%) | Reference |  | 13974 | 1489 (10.66%) | Reference |  |  |
| Secondary plus | 1656 | 196 (11.84%) | 0.91 (0.78 – 1.06) |  | 1225 | 123 (10.04%) | 1.03 (0.86 – 1.24) |  |  |
| Paternal education |  |  |  |  |  |  |  |  |  |
| No formal schooling | 189 | 25 (13.23%) | 0.79 (0.55 – 1.14) | 0.04* | 1218 | 254 (20.85%) | 1.51 (1.32 – 1.73) | < 0.001* | 0.005 |
| Some primary | 221 | 50 (22.62%) | 1.45 (1.13 – 1.87) |  | 1481 | 221 (14.92%) | 1.22 (1.07 – 1.40) |  |  |
| Completed primary | 6631 | 968 (14.60%) | Reference |  | 14588 | 1597 (10.95%) | Reference |  |  |
| Secondary plus | 2742 | 321 (11.71%) | 0.87 (0.77 – 0.99) |  | 1889 | 166 (8.79%) | 0.90 (0.77 – 1.06) |  |  |
| Wealth quintile |  |  |  |  |  |  |  |  |  |
| Q1 (Poorest) | 1577 | 280 (17.76%) | 1.37 (1.13 – 1.67) | 0.001* | 3988 | 569 (14.27%) | 1.39 (1.21 – 1.59) | < 0.001* | 0.053 |
| Q2 | 2325 | 339 (14.58%) | 1.17 (0.97 – 1.41) |  | 4350 | 638 (14.67%) | 1.42 (1.24 – 1.62) |  |  |
| Q3 | 1712 | 249 (14.54%) | 1.21 (0.99 – 1.46) |  | 3247 | 321 (9.89%) | 1.10 (0.95 – 1.28) |  |  |
| Q4 | 2988 | 381 (12.75%) | 1.11 (0.92 – 1.33) |  | 3810 | 396 (10.39%) | 1.13 (0.98 – 1.30) |  |  |
| Q5 (Richest) | 1282 | 142 (11.08%) | Reference |  | 3828 | 336 (8.78%) | Reference |  |  |
| Place of birth** |  |  |  |  |  |  |  |  |  |
| Home | - | - | - | - | 2646 | 423 (15.99%) | 1.24 (1.12 – 1.37) | 0.004 | - |
| Facility | - | - | - |  | 17100 | 1898 (11.10%) | Reference |  |  |
| Trimester of first ANC visit |  |  |  |  |  |  |  |  |  |
| 1^st^ Trimester | 1133 | 151 (13.33%) | 0.87 (0.74 – 1.01) | < 0.001* | 1536 | 183 (11.91%) | 0.89 (0.77 – 1.03) | 0.79* | < 0.001 |
| 2^nd^ Trimester | 6604 | 1006 (15.23%) | Reference |  | 12486 | 1590 (12.73%) | Reference |  |  |
| 3^rd^ Trimester | 802 | 178 (22.19%) | 1.46 (1.26 – 1.68) |  | 2031 | 244 (12.01%) | 0.93 (0.82 – 1.05) |  |  |

*p value for trend

**Participants in Dar es Salaam primarily enrolled from facilities

Table S2. Sensitivity Analysis for DTP3 - Predictors of late (> 128 days of age) or no receipt of DTP3 vaccination among urban Dar es Salaam (n=10,932) and rural Morogoro (n=19,571) infants.

|  | Urban Dar es Salaam | | | | Rural Morogoro Region | | | | p-value for interaction by site |
| --- | --- | --- | --- | --- | --- | --- | --- | --- | --- |
| Characteristic | Total N | Late or no receipt of DTP3  n (%) | Multivariate Relative Risk (95% CI) | p value | Total N | Late or no receipt of DTP3  n (%) | Multivariate Relative Risk (95% CI) | p value |  |
| Total cohort | 10932 | 3439 (31.46%) | - | - | 19571 | 6577 (33.61%) | - | - |  |
|  |  |  |  |  |  |  |  |  |  |
| Infant sex |  |  |  |  |  |  |  |  |  |
| Male | 5578 | 1789 (32.07%) | 1.04 (0.98 – 1.10) | 0.18 | 10406 | 3500 (33.63%) | 1.01 (0.97 – 1.05) | 0.62 | 0.32 |
| Female | 5350 | 1648 (30.80%) | Reference |  | 9165 | 3077 (33.57%) | Reference |  |  |
| Parity |  |  |  |  |  |  |  |  |  |
| Firstborn | 2509 | 784 (31.25%) | Reference |  | 4807 | 1599 (33.26%) | Reference |  | 0.65 |
| 2^nd^-4^th^ child | 5147 | 1625 (31.57%) | 1.11 (1.02 – 1.20) | 0.01 | 8989 | 2936 (32.66%) | 0.96 (0.91 – 1.02) | 0.15 |  |
| 5^th^ or greater child | 668 | 255 (38.17%) | 1.36 (1.20 – 1.55) | < 0.001 | 2978 | 1219 (40.93%) | 1.20 (1.11 – 1.29) | < 0.001 |  |
| Birthweight |  |  |  |  |  |  |  |  |  |
| Low birthweight (<2500g) | 942 | 318 (33.76%) | 1.04 (0.95 – 1.14) | 0.43 | 2713 | 681 (25.10%) | 0.73 (0.68 – 0.78) | < 0.001 | < 0.001 |
| Normal birthweight (≥2500g) | 9990 | 3121 (31.24%) | Reference |  | 16858 | 5896 (34.97%) | Reference |  |  |
| Gestational Age |  |  |  |  |  |  |  |  |  |
| Preterm (<37 weeks) | 1478 | 490 (33.15%) | 1.10 (1.01 – 1.20) | 0.02 | 1861 | 738 (39.66%) | 1.03 (0.97 – 1.10) | 0.33 | 0.37 |
| Term (≥37 weeks) | 6280 | 1793 (28.55%) | Reference |  | 9887 | 3492 (35.31%) | Reference |  |  |
| Size for gestational Age |  |  |  |  |  |  |  |  |  |
| SGA (<10th percentile) | 710 | 218 (30.70%) | 1.01 (0.90 – 1.13) | 0.88 | 1559 | 524 (33.61%) | 0.96 (0.89 – 1.04) | 0.18 | 0.38 |
| No SGA (≥10th percentile) | 6922 | 2018 (29.15%) | Reference |  | 9885 | 3599 (36.41%) | Reference |  |  |
| Maternal age |  |  |  |  |  |  |  |  |  |
| <20 years | 1785 | 638 (35.74%) | 1.33 (1.21 – 1.47) | < 0.001* | 4472 | 1579 (35.31%) | 1.08 (1.01 – 1.15) | < 0.001* | 0.26 |
| 20-25 years | 3204 | 951 (29.68%) | 1.09 (1.01 – 1.18) |  | 5219 | 1764 (33.80%) | 1.06 (1.00 – 1.12) |  |  |
| 25-30 years | 2783 | 784 (28.17%) | Reference |  | 5196 | 1706 (32.83%) | Reference |  |  |
| 30-35 years | 1378 | 388 (28.16%) | 0.95 (0.86 – 1.05) |  | 2725 | 869 (31.89%) | 0.94 (0.88 – 1.01) |  |  |
| ≥ 35 years | 579 | 169 (29.19%) | 0.93 (0.80 – 1.07) |  | 1384 | 458 (34.96%) | 0.90 (0.82 – 0.98) |  |  |
| Maternal education |  |  |  |  |  |  |  |  |  |
| No formal schooling | 655 | 232 (35.42%) | 1.05 (0.94 – 1.18) | < 0.001* | 2175 | 1001 (46.02%) | 1.22 (1.15 – 1.30) | < 0.001* | 0.23 |
| Some primary | 339 | 129 (38.05%) | 1.12 (0.98 – 1.30) |  | 1858 | 764 (41.12%) | 1.17 (1.10 – 1.25) |  |  |
| Completed primary | 7163 | 2200 (30.71%) | Reference |  | 13847 | 4305 (31.09%) | Reference |  |  |
| Secondary plus | 1625 | 388 (23.88%) | 0.84 (0.76 – 0.92) |  | 1213 | 317 (26.13%) | 0.91 (0.82 – 1.01) |  |  |
| Paternal education |  |  |  |  |  |  |  |  |  |
| No formal schooling | 187 | 55 (29.41%) | 0.84 (0.67 – 1.05) | 0.29* | 1197 | 602 (50.29%) | 1.20 (1.13 – 1.29) | < 0.001* | 0.008 |
| Some primary | 217 | 93 (42.86%) | 1.29 (1.10 – 1.51) |  | 1461 | 558 (38.19%) | 1.04 (0.97 – 1.12) |  |  |
| Completed primary | 6495 | 2020 (31.10%) | Reference |  | 14459 | 4684 (32.40%) | Reference |  |  |
| Secondary plus | 2702 | 709 (26.24%) | 0.95 (0.88 – 1.03) |  | 1887 | 503 (26.80%) | 0.97 (0.89 – 1.05) |  |  |
| Wealth quintile |  |  |  |  |  |  |  |  |  |
| Q1 (Poorest) | 1544 | 583 (37.76%) | 1.29 (1.15 – 1.45) | < 0.001* | 3938 | 1537 (39.03%) | 1.37 (1.27 – 1.47) | < 0.001* | 0.01 |
| Q2 | 2276 | 732 (32.16%) | 1.13 (1.01 – 1.27) |  | 4311 | 1779 (41.27%) | 1.44 (1.35 – 1.55) |  |  |
| Q3 | 1672 | 512 (30.62%) | 1.11 (0.99 – 1.25) |  | 3222 | 988 (30.66%) | 1.18 (1.09 – 1.27) |  |  |
| Q4 | 2947 | 772 (26.20%) | 0.99 (0.88 – 1.11) |  | 3769 | 1127 (29.90%) | 1.14 (1.06 – 1.23) |  |  |
| Q5 (Richest) | 1258 | 323 (25.68%) | Reference |  | 3793 | 945 (24.91%) | Reference |  |  |
| Place of birth |  |  |  |  |  |  |  |  |  |
| Home** | - | - | - | - | 2614 | 1203 (46.02%) | 1.25 (1.19 – 1.31) | < 0.001 | - |
| Facility | - | - | - |  | 16938 | 5361 (31.65%) | Reference |  |  |
| Trimester of first ANC visit |  |  |  |  |  |  |  |  |  |
| 1^st^ Trimester | 1108 | 298 (26.90%) | 0.86 (0.78 – 0.96) | < 0.001* | 1517 | 551 (36.32%) | 1.00 (0.94 – 1.08) | 0.19* | < 0.001 |
| 2^nd^ Trimester | 6469 | 2055 (31.77%) | Reference |  | 12350 | 4249 (34.40%) | Reference |  |  |
| 3^rd^ Trimester | 781 | 328 (42.00%) | 1.31 (1.20 – 1.43) |  | 2014 | 667 (33.12%) | 0.95 (0.89 – 1.01) |  |  |

*p-value for trend

**Participants in Dar es Salaam primarily enrolled from facilities
